# Ergot as a Remedial Agent in Epidemic Cholera

**Published:** 1873-07

**Authors:** G. E. Sussdorff

**Affiliations:** Macon, Ga.


ATLANTA

### Medical and Surgical Journal.

Vol. XI.—JULY, 1873.—No. 4.

---

### ORIGINAL COMMUNICATIONS.

---

### ERGOT AS A REMEDIAL AGENT IN EPIDEMIC CHOLERA.

BY G. E. SUSSDORFF, M. D., MACON, GA.

I propose to discuss the subject briefly, and venture the following remarks with hesitation, because, though based upon the strongest and most rational of theories, they yet want the verification of practice. But, having given some thought to the treatment of epidemic cholera, as laid down in the books, and to that also most generally adopted by the profession in this country, and finding nowhere any more satisfactory explanation of the treatment suggested than, in my opinion, can be offered for and in support of my views, I trust I may be pardoned for proceeding at once to discuss the subject by the consideration of the *modus operandi* of ergot in the system.

In "Waring's Practical Therapeutics," we find most satisfactory and reliable information on this point, as follows:

"The action of ergot has been much disputed; but it appears *certain* that it possesses the power of *acting directly* upon, and exciting *contraction* of, involuntary or unstriped muscular fibre. The uterus, especially in the gravid state, is the principal variety of this kind of muscular fibre, and it is on this that its effects are most marked and best known; but we have it also existing in the bladder, the gullet and stomach, the intestinal canal, the bronchial tubes, the ducts of many glands, the iris, and, what is still more important, the middle coat of arteries. We have also in the heart a great involuntary muscle, though its fibres are not of the unstriped variety. Dr. A. Meadows considers it probable that ergot affects the muscular fibre in every one of these structures, in a greater or less degree, though it does not affect these greatly either in the same or in different persons. It is to the action thus exercised on the *muscular coats of capillaries* that is doubtlees due the astringent power that ergot displays in cases of hemorrhage, and the same fact explains in a measure its power as an emmenagogue."

These views are endorsed by many who have had extensive experience in its use, and we must take for granted, in this paper, the truth of these observations, for it is upon these facts that we propose its administration in the treatment of epidemic cholera.

Bearing these facts in mind, let us proceed briefly to examine into our knowledge respecting the anatomical character of epidemic cholera.

So far as can be ascertained, there are no "appreciable lesions which seem commensurate with the gravity of the disease."

The microscope shows the absence, more or less, of gastric and intestinal epithelium, which seems the only constant lesion.

There is often submucous congestion, but no signs of inflammatory action.

The various organs of the body, as a rule, present no abnormal appearance beyond an unusual amount of paleness, and marked deficiency of liquids.

"The morbid appearances are not in themselves considered sufficient to give an explanation of the symptomatic phenomena, nor do they give us any light upon the pathology of the disease."

Each case presents its own individual lesions, when any exist, and may be considered in a measure as accidental.

The disease does not *necessarily* involve any serious damage of solid parts, as shown by the rapid recovery in some cases in which the disease is promptly arrested.

The blood, however, presents more decided and constant alterations. It is notably deficient in albumen, fibrin, and all the constituents of serum." It loses its watery portion to a great degree, giving it a greater consistency, making it thick and dark-colored. The whole train of phenomena in this disease are doubtless due to this condition of the blood alone.

In the pathology of this disease, the first appreciable event is the transudation into the alimentary canal, and upon it the usual symptomatic phenomena follow, to a greater or less extent.

Now, it is a settled fact that there is a specific poison which alone can produce this disease, but *how* it acts within the system is a matter yet to be satisfactorily accounted for. Some assert that this poison acts primarily, as a local cause, *within* the intestinal canal; and others tell us it is absorbed into the circulation by means of the stomach, lungs and skin. This latter theory seems the more popular at present.

One writer, Robin, attributes the pathological changes to an isomeric modification of the coagulable constituents of the blood plasma, in consequence of which they part with the water entering into their composition, and lose the power of appropriating water.

Perhaps I may be allowed to express my favor of the theory which is as follows:

The " materies morbi," being absorbed into the blood, is carried to the *nerve centres* governing the circulation, and especially to those governing the tonicity and physiological action of the coats of capillaries, throughout the *whole economy.* The poison here works the changes which sooner or later produce relaxation of these capillary coverings to such an extent that there ensues more or less transudation of the watery part of the blood—principally into the intestines, though it may take place within any or all the cavities of the body. The capillaries of the skin seem most affected after those of the intestinal canal, as evidenced by excessive sweating.

This poison, then, acts by causing a relaxation of the coverings of the capillaries, and, to a greater or less extent, of all organs containing involuntary or unstriped muscular fibre; and whether you accept this theory or not, in explanation of the way this poison acts, one thing remains which is important, and which seems almost certain: that if you can *prevent* this transudation, or control it promptly after it first takes place, you generally succeed in arresting the disease, and without sequelæ. Most writers have given this condition the name of transudation, but it may without impropriety be called a hemorrhage, as it seems to partake of these characters specially.

I said I would be brief, and though I might enter more extensively into a discussion of the whole subject, I will not do so, but endeavor to give these outlines sufficiently bold and plain to have my ideas understood.

In ergot we have an agent of known and *immediate* power in producing *contraction* of involuntary or unstriped muscular fibre. By its influence we have the power of arresting profuse hemorrhages from the uterus, and we also can control the hemorrhages from smaller arteries, as well as capillary oozings *in all parts of the body.* The action of ergot, as a prompt and powerful astringent, is known to all practitioners, and I need not enumerate instances illustrative of it. Ergot also has a stimulative action upon the *heart,* when this organ is in a relaxed and flaccid condition. Let us not forget the action of ergot as given in the first part of this paper.

In America, the most successful plan of treatment has been by preventing or contracting this transudation or hemorrhage. It seems almost certain that if you can stop the premonitory diarrhœa, you have prevented the disease, and it seems even more certain that, when this hemorrhage or transudation into the bowels has been established, if you can arrest *it* before too great changes have been wrought in the blood-plasma, you may generally expect a complete recovery. The retention in the blood of this poison does not appear to give any more trouble—its effects are *transient.*

The great object, then, seems to be to find some agent which will be prompt in producing a powerful astringent influence upon the whole circulation, but especially upon the capillary system.

*I believe ergot capable of inducing this condition more effectually than any other remedy known to us,* and that it will prove in this way a remedy of the utmost importance in the treatment of epidemic cholera. If it controls hemorrhages of the *blood,* it is likely to control hemorrhages of *serum* of the blood. It probably acts as an astringent, principally by its influence upon the great nerve *centers,* by inducing contraction of the unstriped muscular fibre, wherever it exists, but especially upon that in the capillary system, overcoming the general relaxation of these parts and restoring them, in a great measure, to their normal condition. It seems to me ergot is likely to do good in the condition of *collapse*, by contracting the calibre of *larger blood vessels*, and giving power to the heart to propel the thickened blood, and at the same time allowing the absorption of water, into the circulation.

These views are based entirely upon *theory*. I have been unable to put them into practice myself, and I offer them to the profession for what they are worth, with the hope that this agent will prove all I expect. I might add that the best way, in my opinion, to use ergot, as a rule, will be by the hypodermic method, in half-grain doses of ergotine, frequently repeated, until some benefit is experienced.

This remedy may be used at any stage of the disease, from the first premonitory diarrhœa to the last condition of collapse. If it can be administered early in the disease it will likely give better results, while the circulation of blood is still sufficiently active to carry it to the nerve centers. Yet it is likely to do much good even in the cyanosed condition of collapse.

The administration of ergot can do no harm if it do no great good, and I know of nothing contraïndicating its use. It will not interfere with the treatment as generally adopted by the profession at the present time.

Not having an opportunity of testing these views, I can offer nothing more upon the subject for the present, but trust those who may meet with the disease will give the remedy a thorough and faithful trial.

---

THE ODOR OF THE BREATH IN DIABETES MELLITUS.—M. Guéneau de Mussy, in the *Gazette Hebdom.*, July 19, says that for several years he has noticed that patients affected with diabetes have a sour breath that smells somewhat like alcohol, or like the heavy breath of the habitual drunkard. In many cases he was able to diagnose the disease from this odor of the breath, and he also noticed that with the decrease in the amount of sugar in the urine the odor disappeared from the breath.—*Berliner Klinische Wochenschrift*, 1872.

